# Changes in Body Composition and Motor Fitness of Young Female Volleyball Players in an Annual Training Cycle

**DOI:** 10.3390/ijerph20032473

**Published:** 2023-01-30

**Authors:** Adrian Sieroń, Aleksandra Stachoń, Jadwiga Pietraszewska

**Affiliations:** Department of Biological and Medical Basis of Sport, Wroclaw University of Health and Sport Sciences, Paderewskiego 35, 51-612 Wroclaw, Poland

**Keywords:** body composition, motoric tests, periodization

## Abstract

Background: Modern volleyball requires a high level of motor preparation, specific body build and optimal body composition. Objectives: The aim of this study was to determine changes in body build, body composition and motor skills in young volleyball female players during an annual training cycle. Methods: The research group was comprised of 36 female athletes aged 14–16 years, who were monitored throughout a whole season using a longitudinal study design. Body composition was estimated by bioelectrical impedance analysis. Motor fitness was assessed with the following tests: vertical jumping tests (based on one-handed and two-handed reach, standing vertical jump and running vertical jump), standing long jump and 2 kg medicine ball throw. Measurements were carried out at the beginning of the preparatory period, after its completion, in the middle of the start period, at the end of the start period and during transition periods. Results: The study showed significant changes in body composition and motor fitness level during the annual training cycle. These changes differ in subsequent periods of the macrocycle. The most pronounced changes occurred after the preparatory period and concerned increases in fat-free mass, total water content and cell mass. A significant reduction in fat content was also noted at this point. Conclusions: Systematic monitoring of morpho-functional changes in young female volleyball players over extended periods provides them a chance to maintain their optimal fitness level.

## 1. Introduction

Optimal distribution of training stimuli in the right order and time is essential in preparing an athlete. The periodization process, the purpose of which is to build an athlete’s highest sports level, consists of changes in intensity, volume and frequency of training loads [1]. Introducing various types of training to complement specialized volleyball training can help prevent injuries and improve lower limb stability [2].

The schedule of matches in league competitions determines the structure of training periods, including preparatory, starting and transition periods. The preparatory period aims to shape the functional and technical basis for future special training. The main goal of this period is to increase potential and accumulate reserves for future sports competitions. This can be achieved by a high share of comprehensive loads, which then decrease in favor of targeted and specialized exercises [3]. The starting period is the stage where microcycles are based on activities that include special exercises. The primary goal at this stage is to increase special preparation level and use it effectively in competitions. The transition period aims to provide an athlete with physical and mental rest after the competition loads and prepare them for the next season [3]. During this period, the training structure should include learning and improving techniques, individual tactics and selected issues of team tactics. 

Volleyball is a discipline in which a training cycle spreads over a long period, which makes it necessary to use special training measures, through which it will be possible to maintain fitness throughout the entire annual cycle. Another factor that should be considered in training is the relationship between the somatic and functional spheres, which affect the sports level of male and female players. Pastuszak et al. [4] indicated that tissue relationships and somatic features are related to level of league games and practicing this discipline professionally. Therefore, selecting people with a specific somatic build for this discipline is essential. The right body structure is one of the elements supporting achieving a high sports level. Numerous authors provide anthropometric characteristics of professional and amateur volleyball players in different countries [5,6,7,8]. The results of these studies indicate a certain differentiation of players depending on their sports level. However, in a given population, volleyball players stand out from other athletes and non-training people by definitely stronger development of length features and slenderness of their build. It should be remembered that, in the case of this discipline, there are also differences in body build of male and female players due to their position on the field [6,9]. Their role justifies this during a game. Players with lower body height and lower limb length are characterized by better effectiveness in defensive actions (libero). On the other hand, very well-developed body length features enable more effective play over the net, both defensively (block) and offensively (attack). 

Moreover, young female volleyball players have a specific physique compared to their peers who do not train, which should be considered as the effect of both selection and specialized training [10]. Young volleyball players of both sexes are characterized by above-average values of height features and a significant slender build. Anthropometric characteristics of young volleyball players define this group as tall people with a low percentage of body fat. The somatotype of young volleyball players is described as ecto-mesomorphic [11,12]. As in the case of senior players and young players, there are significant differences in body structure due to court position. The game’s specificity often forces the team’s shortest players to specialize in the libero position. Young players playing in attacking and middle positions are distinguished by higher body mass conditioned by their considerable height and a more significant amount of fat-free body mass. This is justified by implementing specific tasks and goals of individual players on the court [11,13]. 

Motor fitness level, which is conditioned by many factors, including morphological factors, is extremely important for an athlete’s efficiency [14,15,16,17]. Vuleta et al. [18] noted significant differences between players of different levels in terms of body height, body weight, attacking jumps and blocking jumps.

Changes in body composition as a result of training loads also translate into improved functional characteristics of athletes, resulting in better performance and motor fitness [19]. A relationship was found between level of body components and motor performance among female volleyball players: athletes with less fat presented better results in the agility and jumping test and athletes with higher fat-free body mass had better long jump results [20,21,22,23]. Similar links between jumping ability and tissue components in senior female volleyball players were found in previous studies [6,24]. Studies conducted among 13–16-year-old female volleyball players showed a high correlation in the results of jumping and strength tests with the majority of anthropometric features [25].

Nikolaidis et al. [26] noted significant differences in fat and fat-free body mass percentage among Greek female volleyball teams: top-level athletes were characterized by less body fat, more muscles and slenderness. Similar variability is described by other authors [27,28]. 

Tracking changes in motor performance of athletes in the context of changing body composition may consequently contribute to improving the results obtained during competitions [29,30,31]. Early identification of athletes experiencing a decline in their sports performance creates an opportunity for coaches to reorganize the intensity and volume of training. Lack of early diagnosis of overtraining can have negative consequences for a particular athlete and the entire team [32]. Monitoring the morphological effects of training also enables eliminating changes in body composition that could be unfavorable to the body’s performance and increase risk of injury [33]. An optimal amount of adipose tissue affects, among others, the ability to regenerate after training, hormone production and thermoregulation [30]. Researchers indicate the need to monitor body composition of athletes’ bodies throughout the training cycle. The results of such studies provide valuable information about the body’s adaptability to applied training loads. This is especially important for young athletes who are undergoing dynamic development processes. 

Despite the importance of these problems, few papers describe these issues concerning the entire macrocycle. Most publications concern cross-sectional studies of the body composition of female volleyball players at a very high level [28,34,35]. They show that level of individual body components, and, above all, ratio of muscle mass to fat, can play an essential role in the ability to take offensive and defensive actions and indirectly determine their effectiveness. Requirements based on physical predispositions in professional games are increasing because their shortcomings must be compensated by other attributes (experience, anticipation or volleyball intelligence). Research confirms that competition at the highest level is associated with optimal somatic predispositions, which include, among others, percentage of fat, amount of fat-free mass and ratio of extracellular mass to body cell mass (ECM/BCM) [36].

It is vital for a coach to monitor multifaceted morpho-functional changes in athletes during the entire macrocycle because it enables them to verify training loads on an ongoing basis in the event of undesirable effects and to modify training in the next macrocycle. However, it involves much effort and commitment from many people. Therefore, most of the previous publications concern shorter training periods or are limited only to assessment of somatic changes [37,38,39,40,41]. The results of the few longitudinal studies on morpho-functional changes in the entire annual training cycle among volleyball players indicate specific trends. Still, they are rather diverse in relation to gender, age group and sports level [42,43,44,45,46,47]. 

Considering the small number of studies and ambiguous results, the study aimed to assess changes in body composition and motor fitness level of young female volleyball players in an annual training cycle. Implementation of the goal will enable verification of the training process for young personnel in terms of maintaining the appropriate level of fitness preparation, which, in turn, should contribute to improvement in the results obtained during matches. 

## 2. Materials and Methods

### 2.1. Materials and Study Protocol

**Figure 1:**
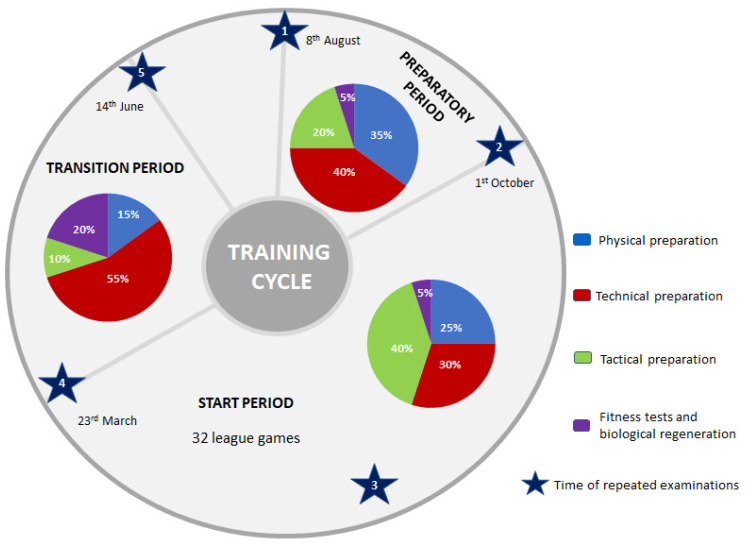
The structure of the macrocycle, considering training characteristics in subsequent periods.

The longitudinal study involved 36 female volleyball players aged 16.0 ± 1.4 who train at the academic sports club (AZS AWF Wroclaw) for 4–6 years. Measurements were carried out 5 times: (1) at the beginning of the preparatory period, (2) after the preparatory period, (3) in the middle of the starting period, (4) at the end of the starting and (5) transition periods. The research protocol covers a full annual macrocycle lasting from August to July of the following year. The scheme of the training cycle with marked test periods is shown in Figure 1.

The participants of the study were female players representing one sports club. The inclusion criteria included: training for at least 4 years, continuous training during whole macrocycle, no injuries in the period preceding the study, no special diets and 14–16 years of age. The condition for exclusion was interruption of training and injuries or diseases that prevented the examination. All initially qualified female athletes participated in subsequent measurements and tests; none of them had exclusion conditions.

During the preparatory period (August–September) the group participated in a total of 44 training units lasting 90 min each. The total time allotted for this training period was 3960 min. In addition, as part of preparations for the tournament, the group played 3 friendly tournaments. Physical preparation during this period included: general strengthening exercises, exercises with stability balls, exercises with rubber bands, initial strength adaptation training, preventive exercises, general endurance exercises, speed, jumping, agility exercises, etc., functional exercises, yoga and warm-ups in the form of stretching, games and other activities. Technical preparation included basic elements of volleyball technique used in warm-ups and serves, taking possession of the ball, play style, attacks, blocks, defense and securing the game. The scope of training measures in terms of tactical preparation concerned small games and task games in 3–6 player configurations and sparring matches. Motor tests and biological regeneration were the other means used during this period. 

In the period from 1 October to 23 March, the players participated in a total of 149 training units lasting 90 min each. The total time allotted for this training period was 13,410 min. During this period, the players participated in games, playing a total of 32 league matches (Figure 1). 

The transition period lasted from 24 March to 14 June. The group participated in a total of 44 training units lasting 90 min each. The total time allotted for this training period was 3960 min (Figure 1).

Repeated examinations included anthropometric measurements, motor test score measurements and body composition analysis. 

### 2.2. Anthropometric Measurements

Height measurements were conducted using a GPM Anthropological Instruments anthropometer (accuracy 0.1 cm). Body weight was measured using an electronic scale with an accuracy of 0.1 kg. Body mass index (BMI) was calculated based on height and weight. 

### 2.3. Body Composition Assessment

Body composition was estimated by the bioelectrical impedance (BIA) method using BIA-101 Anniversary Sport Edition analyzer and Bodygram^®^ Plus Software v.1.0. (Pontassieve, Italy) The tests were performed following the rules and procedures applicable to this method. Body composition measurements were performed 5 times, each time in the same phase of an athlete’s menstrual cycle. The following components of body mass, expressed in kilograms and as a percentage of body mass, were used for analysis: fat-free mass (FFM), total body water (TBW), body cell mass (BCM), fat mass (FM).

### 2.4. Motor Fitness Assessment

To assess the motor efficiency of the study participants, the following motor tests were used (after a 15 min warm-up): jumping without a run-up (three attempts were made and the best result was used for analysis); jumping with a run-up (the leaf device was set at a distance of 1 m from the net, three attempts were performed and the best result was used for analysis); medicine ball throw (three attempts were made and the best result was used for analysis; the 2 kg ball was held with both hands at chest height, feet hip-width apart, parallel and in front of the throw line; after performing a swing with the ball, bending the legs at the knees and slightly tilting the torso backwards, a study participant energetically threw the ball in a gentle arc as far forward as possible); standing long jump (three attempts were made and the best result was used for analysis). Long jump and ball throw distances were measured with a measuring tape to the nearest 1 cm.

The jumping tests were performed using the Vertical Jump Trainer. The one-handed and two-handed reach as well as vertical jump height with and without run-up were measured. Then, the differences between vertical jump height without run-up and two-handed reach as well as vertical jump height with run-up and one-handed reach were calculated, which enabled estimating jumping abilities. Jump Trainer is a device that consists of a base and a leaf module and measures standing reach and vertical leap reach. Measurement is completed by tilting the leaves. 

### 2.5. Ethical Issues

This study was conducted in accordance with the requirements stipulated in the Declaration of Helsinki and was approved by the Ethics Committee of Wroclaw University of Health and Sport, Poland (2/2020). The women provided their voluntary consent to participate in the study. Before each test, they were familiarized with the procedures and contraindications for measurement. 

### 2.6. Statistical Analysis

Statistical analyses and graphs were performed in the Statistica 12.0 program (TIBCO Software Inc., Palo Alto, CA, USA). The distributions of the analyzed variables did not differ significantly from the normal distribution. Analysis of variance with repeated measures was used to assess differences for all the analyzed features. A post hoc test for multiple comparisons (least significant difference test of Fisher) was also performed. This test evaluated the significance of the differences between the means in the subsequent stages of the macrocycle. 

## 3. Results

Analyzing changes in basic somatic features, body composition and results of motor tests, significant differences were found for most of them between individual tests in the annual training cycle.

### 3.1. Changes in Basic Somatic Features and Body Composition

In the case of body height, a significant increase in this feature was noted throughout the entire macrocycle (Table 1). Body weight after the preparatory period slightly decreased; increases in this feature were noted in subsequent tests, with significant changes occurring after the start and transition period (Table 1). When analyzing the BMI values, a significant difference was found only between the middle of the starting period and the end of the macrocycle, where there was an apparent increase in the massiveness of the build (Table 1). 

**Figure 2:**
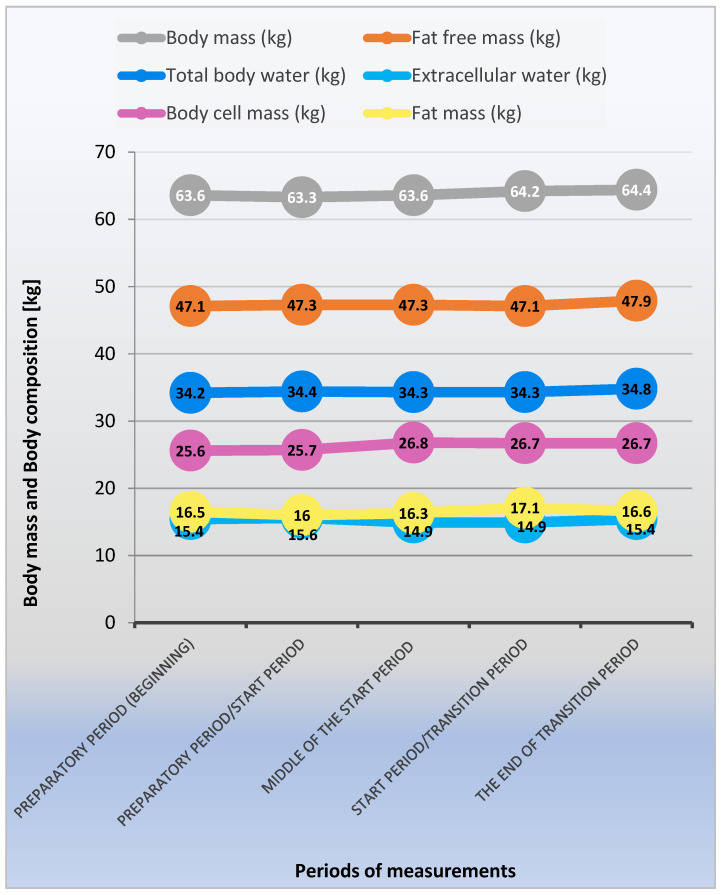
Changes in body composition in successive periods of measurements (1–5) during a macrocycle.

Tissue component changes vary (Figure 2). Fat-free mass expressed in kilograms does not change significantly from the first examination to the end of the starting period. Only in the fifth test, at the end of the transition period, a significant increase in this component was found (Table 1). In the case of percentage of fat-free mass, a fluctuating course of changes was found in subsequent training periods. Still, statistically significant differences were noted at the end of the starting period, when the content of this component in body weight decreased. However, in the transition period, there was a return to the values from the beginning of the preparatory period (Table 1). Total water content showed similar change tendencies to fat-free mass (Table 1). In the case of percentage of water content, a diversified direction of changes during the training cycle was observed. After a slight increase in the water content after the preparation period, there was a significant decrease in its percentage throughout the entire starting period. After the transition period, the content of water increased to the level from the beginning of the study (Table 1). 

Cell mass expressed both in absolute and percentage values after the preparation period does not change significantly. Then, after two months of training and competitions, a significant increase and stabilization are observed until the end of the competition period. There were no significant changes in cell mass during the transition period (Table 1). 

In the case of fat mass, a statistically significant decrease in the mean value after the preparation period was found. Then, during the starting period, the volleyball players tend to increase this component, with a significant increase between the middle of the starting period and its end. The effect of these changes is the highest value of fat mass at the end of the starting period. At the end of the macrocycle, test 5 showed a significant decrease in the value of this feature. A similar course of change was noted for percentage of fat in body weight (Table 1). 

### 3.2. Changes in Motor Fitness

Analysis of jumping without a run-up showed significant differentiation in results in subsequent stages of the macrocycle. After the preparatory period, jumping ability definitely improved. Then, in the first half of the starting period, it deteriorated, and then the results improved again in the second part of the starting season. After the transition period ending the study, the results decreased once more (Table 2). In the case of jumping with a run-up, an improvement in results was observed from the beginning of the preparatory period to the end of the starting period. In the tests carried out at the end of the starting period, the players obtained the best result in this test in the entire macrocycle. In the transition period, deterioration in the results of jumping with a run-up was observed (Table 2). The changes are insignificant in the 2 kg medicine ball throw test after the preparatory period. However, in the starting period, a significant improvement in the results of this test was noted (Table 2). The length of long jump without a run-up increased significantly after the preparatory period and after the first half of the competition period. Then, the results stabilized until the end of the studied macrocycle (Table 2).

## 4. Discussion

Analysis of changes in body height during the macrocycle in the young female volleyball players indicated that they had already passed the stage of the most dynamic development of somatic features and were in the late adolescence stage. Their average body height was similar to that of young players described in previous publications [13,48,49]. Although female players in senior teams achieve higher values of body height [5,50,51,52,53], it should be assumed that the tested female athletes will achieve slightly higher values for this feature in subsequent years. It should also be pointed out that the greater body height of female volleyball players is largely the result of morphological selection [25,54,55,56]. Comparison of examined female athletes with their non-training peers showed that their body height is definitely higher [57]. 

The studied group presented the correct weight–height relationship assessed with the BMI index, which is related to the high physical activity of the players. Other researchers also made similar observations [27,49,58].

The study’s main aim was to assess changes in body weight and its components in the annual training cycle. The observed changes in body weight during the macrocycle are mostly reflected in the literature. The examined volleyball players showed a decrease in body weight after the preparation period and a gradual increase in weight until the end of the analyzed macrocycle. Lehnert et al. [59] and Stamm et al. [60] reported similar results in relation to the preparatory period. Rousanoglou et al. [44] and Pavlík et al. [40] showed weight gain in female volleyball players during the competition period. However, different results are presented by Hyatt and Kavazis [61]: they observed a constant decrease in body weight in a university team during the starting period and a change in this tendency only after the league matches. 

It should be remembered that body weight, as a heterogeneous feature, does not provide information on amount of fat and muscle tissue or general hydration and body condition. It is also related to changes in body height. Therefore, an analysis of changes in its components and weight to height index is a necessary supplement. In particular periods of the macrocycle, the studied female athletes showed slight fluctuations in their body mass. They slimmed down after the preparatory period, which was previously confirmed [59,61]. According to other authors, BMI value increases in the initial period and fluctuates in the following stages of the macrocycle [37,41]. Since BMI does not provide information on body composition in athletes [62,63], analyses of individual components of body weight seem essential. 

The fundamental component of body mass is cell mass, which includes metabolically active somatic cells, mainly muscle tissue [64] and potassium-rich internal organs [65]. When assessing the level of this component in the studied female volleyball players, it was found that it is comparable to the results of female players from the top team from the Czech league [36]. However, the literature describes groups of highly qualified female athletes with a higher content of cell mass [34]. Observed changes in cell mass during the macrocycle indicate an increase in absolute values of this feature during the preparatory period (but a slight decrease in percentage values), followed by a significant increase in the first half of the starting period. It can be assumed that such reactions of the body were influenced by an increase in the volume of strength exercises in the starting period.

The next analyzed component was fat-free mass. After the preparatory period, an increase in this component was noted in the studied group. Stojanović et al. [41] also found an increase in this component after 4 weeks of preparatory training. On the other hand, Buśko and Lipińska [37], after preparations for league games, noted an insignificant decrease in fat-free mass among female volleyball players. Interpreting the results of the current study, it should be assumed that the predominance of strengthening and functional exercises in this period resulted in an increase in musculature and, consequently, fat-free mass. There was a clear increase in this component at the end of the transitional period. Similar results were reported by Stanforth et al. [39], who tracked changes over the two subsequent macrocycles. In the studies of the National Collegiate Athletic Association Division I on female volleyball players, a gradual increase in fat-free body mass was noted until the middle phase of the starting period and a decrease in this component until the end of the transition period [38]. 

Analysis of percentage values of fat-free mass shows more dynamic changes in this component. After a slight increase in the percentage of fat-free mass after the preparatory period, a significant decrease was noted until the end of the starting period. Considering the constant level of absolute fat-free mass in this period, this result should be interpreted as the effect of reducing the fat mass among the tested athletes. 

Fat tissue performs important functions related to proper functioning of the human body [66]: it plays a vital role in the process of thermoregulation and protection of internal organs; it is also a solvent of essential vitamins. Fat is also the second, after carbohydrates, energy substrate for working muscles. Both its excess and deficiency can cause metabolic disorders. Research confirms that female volleyball players are among the athletes characterized by a low level of fat [67,68]. This feature distinguishes highly qualified young female volleyball players from representatives of other sports disciplines [54]. Analyzing the body fat of the examined female players, values similar to those of other volleyball teams were found [6,27,28,58,69]. At the same time, it should be noted that players with lower body fat usually represent a higher level of fitness [24,26,34,36]. 

Observing changes in amount of adipose tissue and fat distribution has become one of the basic elements of athlete preparation [30]. Due to variability in fatness and the possibility of its reduction under the influence of training, it is worth paying attention to the diverse response of the body to loads in the following periods of the macrocycle. After the preparatory period, this component decreased significantly. It should be recognized that this is the result of training loads with a hefty dose of endurance exercises. In subsequent tests, there was an increase in fat content, with a significant increase at the end of the starting period. After the transition period, the latest test indicated a significant decrease in the amount of fat in the volleyball players. Similar trends as in the present study were also demonstrated by other authors [40,41,59]. On the other hand, Rousanoglou et al. [44], examining teenage female volleyball players after the preparatory period, found a significant increase in this component. Häkkinen [42] pointed to lability of fat during the competition period. Most studies show a decrease in this component during league games [9,61,70,71]. However, some authors report an increase in adipose tissue in this period of the macrocycle, similar to the female volleyball players in current study [38,72]. It cannot be ruled out that increase in fat in the starting period is related to stress and emotions accompanying the players during league games, which is compensated by, e.g., an unhealthy diet rich in sugar and fat. On the other hand, longitudinal studies by Stanforth et al. [39], covering several macrocycles, indicate varied changes in this component in individual players, which attests to the need to individualize training loads. 

Water creates the right environment for various metabolic processes in the body. In addition, water regulates body temperature during physical activity and increased external temperature, thus performing a thermoregulatory function [73]. In the tested volleyball players, water constituted approximately 54% of their body weight. Compared to less physically active women, these values are definitely higher [36,74]. A high proportion of water is characteristic primarily of elite female volleyball players [34,36]. The representatives of Serbia during the Olympic Games in Rio de Janeiro had an average water content of 47.85 kg, which was over 63% of their body weight [75]. In present study, a sharp increase in amount of water occurred only in the transition period. 

On the other hand, in percentage values, apparent increases were observed after the preparatory period and in the transitional period. In the starting period, the percentage of water in body weight decreased. It can be assumed that this results from training loads and lack of control over players’ consumption of the right amount of fluids. Impaired fluid intake of athletes during the start period affects tissue composition and body water content [76]. In volleyball, the nature of training work varies due to the loads in a given period. Increased work intensity determines an increase in heat production and water loss [77]. Particular attention should be paid to increasing fluid intake in such conditions to bring the body to a balanced state [78]. If this aspect is neglected, the body’s hydration will be disturbed. 

Stojanović et al. [41], at the end of the 4-week preparatory period, found an increase in total water content by more than 3% among leading female Bosnian volleyball players. In turn, the results of Buśko and Lipińska [37] indicate slight differences in amount of water in absolute terms in the entire macrocycle, similar to the present findings. The results obtained in the current study indicate the highest water content at the end of the transition period. The reasons for this seem to be the training measures used during this period, reduced emphasis on physical preparation and a lack of matches and adequate rest. Similar results in players of various team sports games, including volleyball, were found by Silva et al. [79]. 

While playing volleyball, there are several complex technical–tactical activities that consist of many individual elements: serving, receiving serves, playing, attacking, blocking, defending, etc. Due to various actions and cyclical changes in a team’s positioning, volleyball requires high motor activity. Volleyball is dominated by speed–strength features (speed, jumping ability, jumping endurance and strength) and coordination features (agility, spatial orientation and kinesthesia). To assess the motor skills that determine effectiveness of young female volleyball players during a game, a set of fitness tests was used. 

Two types of vertical jumps, evaluating one-handed reach while jumping with a run-up and two-handed while jumping without a run-up, were performed. When performing an attack or serve, the most common form used by volleyball players is a jump preceded by a run-up. This way of moving allows athletes to achieve higher jump values and may indirectly contribute to better efficiency in the above activities [80]. During the tests, the young volleyball players were characterized by a one-handed reach in the range of 274.9–283.6 cm, which means jumping abilities during running of 49.1–56.9 cm height. On the other hand, two-handed reach without a run-up is a test showing similarities to execution of a single block. This element is often performed among intermediate groups after displacement and in groups. The nature and rules of the game make it one of the most common forms of players’ movement during matches (except for libero players). The actual jumping abilities in this element were in the range of 38.9–45.8 cm height. Comparable results for jumping with a run-up in the preparatory period were obtained by Tsoukos et al. [81] in a group of young female volleyball players. In the case of standing vertical jump, the players in the study mentioned above jumped much higher. 

Analysis of changes in the subsequent stages of the macrocycle showed a significant improvement in the results achieved in the above tests after the preparatory period and their stabilization or increase until the end of the starting period. The jumping level achieved by the players was consistent with the assumptions made for these stages. Research by Bilica and Selçuk [82] showed development of lower limb muscle strength and improved jumping ability after a 10-week training session. Rousanoglou et al. [44] also indicate that progression in jumping ability is the result of enhancing the ability of volleyball players to produce more power in a limited time. Furthermore, in the transition period, a slight decrease in the results of the jumping tests was observed. This result seems evident due to reduction in training measures used in terms of physical preparation. The most significant improvement in the results of the tests in the preparatory period is because the volume of physical exercise in this period was 20% higher than in the mesocycle ending the macrocycle. 

Diagnosis and assessment of changes in motor efficiency in tests assessing the dynamic strength of the lower and upper limbs of young female volleyball players are critical in preparing and maintaining readiness of volleyball players for changing effort in the macrocycle and may determine effective implementation of playstyle during a match. In order to determine dynamic strength of the muscles of the shoulder girdle and arms, 2 kg medicine ball throw was used. This throw can be classified as a speed–strength exercise due to the significant speed of the performed movements and involvement of many muscle groups. After the preparatory period, there was no improvement in the results of this test. However, during the starting period, the results were constantly growing. As a result, the players achieved the best results at the end of the starting period. It can be stated that it was the effect of the increase in strength resulting from the time and impact of training and competition loads. These results confirm the observations of Tessutti et al. [69].

The obtained data are consistent with previous studies that demonstrated the ability of athletes to improve motor fitness during controlled training [83]. It should be noted that motor fitness requires an optimal balance between training loads and recovery time [84]. This balance can be maintained thanks to a properly structured program [85].

In analysis of standing long jump, a dynamic improvement in jump distance was noted from the beginning of the preparatory period to the middle phase of the starting phase. The training used at that time significantly improved the jumping distance of all the volleyball players, proving that the training loads were selected correctly. However, individual differences in progression of the results indicate the legitimacy of individualization. Marked improvement noted in the initial period of the macrocycle is probably due to a lack of systematic training before the start of the preparatory period and then adaptability to the applied training loads by the nervous system and muscles [86].

The above-described changes in body composition and changes in the results of motor tests are a good illustration of the connections between the somatic and motor spheres. A review of the literature confirms that correlations with body build characteristics are clear for many components of motor fitness. Nikolaidis [23] found that volleyball athletes with high BMI and body fat scored lower in fitness tests. Gabbett et al. [87] also indicate that too much fat in the body is associated with decreased performance and hinders locomotion on a volleyball court. Acar and Eler [88] found a positive correlation between the results of the vertical jump and long jump attempts and body weight in their study of teenage female volleyball players. Similar results are presented by Aslan et al. [89]. In turn, Aouichaoui et al. [90], in their studies of adolescent athletes, showed a negative correlation between jumping ability and height and weight, as well as BMI.

Muscles allow for generating the right force to overcome resistance and perform motor tasks. Their level of development is, therefore, an indicator of many elements of motor fitness. According to Martín-Matillas et al. [28], high content of muscle mass characterizes the best volleyball players. Mielgo-Ayuso et al. [9] describe positive correlations between jumping tests and thigh and lower leg circumference, reflecting lower limb musculature development. Ćopić et al. [24] indicate that, apart from muscles, other components of body composition may be significant predictors of effectiveness in jumping tests. Researchers emphasize that appropriate tissue relationships can contribute to improvement in sports performance. Morrow et al. [91], analyzing the results of a women’s volleyball tournament, found that lower fat content was associated with higher places of participating teams. In other studies, Kutáč and Sigmund [36] showed that, at the amateur level, the right body height and proper body tissue relationships are important factors influencing the final sports result in volleyball. In the study of Silva et al. [79], among representatives of team sports games, a positive relationship was found between amount of water and results of strength and jumping tests. According to the authors, greater cell hydration is a factor causing intensification of anabolic processes, leading to improvement in muscle strength. 

When analyzing the morpho-functional changes in female volleyball players, they are related to changes in training loads. Diverse physical loads are reflected in changes in body composition, which may affect athletes’ motor fitness levels.

## 5. Limitations of the Study

The fact that all the players come from the same club may limit the generalizability of the study results. Basing the study on a larger sample could generate clearer differences between compared periods. However, this approach also has some advantages. The players of the same club share a similar model of physical preparation and strength development, thanks to which training was not a factor differentiating them. From a coach’s point of view, the study provided valuable information for evaluating the effects of applied training in a specific group of volleyball players. A limitation in interpreting the study results may also be the lack of detailed interviews regarding caloric intake and diet nutrients. A general dietary interview was conducted with the players, which indicated that they had not used any special medical or slimming diets before or during the research. It seems that diet is not a factor differentiating the examined women. 

## 6. Conclusions

The current study showed significant changes in body composition and motor fitness level of young female volleyball players during an annual training cycle. Changes in amount of individual body tissue components differ in subsequent periods of the macrocycle because the nature of training is different in individual mesocycles. The most pronounced changes occurred after the preparatory period and concerned increases in fat-free mass, total water content and cell mass. A significant reduction in fat content was also noted at this point. 

The significant improvement in motor fitness after the preparatory period noted in the current study is consistent with the training assumptions for this period, where the emphasis was placed on technical and motor activities. Systematic monitoring of morpho-functional changes in young female volleyball players over extended periods provides them a chance to maintain their optimal fitness level. 

## Figures and Tables

**Table 1:** Statistical characteristics of the basic somatic features and body composition of female volleyball players in subsequent periods of the macrocycle.

Variables		Examinations				
		1	2	3	4	5
Body height (cm)	M	172.19 ^efg^	172.23 ^bhi^	172.91 ^bcej^	173.10 ^cfh^	173.14 ^gij^
	SD	6.87	6.89	6.84	6.91	6.94
Body mass (kg)	M	63.61 ^g^	63.28 ^hi^	63.61 ^j^	64.17 ^h^	64.45 ^gij^
	SD	10.31	9.92	9.54	9.62	9.59
Fat-free mass (kg)	M	47.12 ^g^	47.30 ^i^	47.28 ^j^	47.14 ^d^	47.88 ^dgij^
	SD	5.07	5.28	5.06	4.69	4.81
Fat-free mass (%)	M	74.73 ^f^	75.27 ^h^	74.74 ^c^	73.96 ^cdfh^	74.74 ^d^
	SD	5.30	4.71	4.82	4.86	4.85
Total body water (kg)	M	34.24 ^g^	34.40 ^i^	34.30 ^j^	34.33 ^d^	34.79 ^dgij^
	SD	3.21	3.26	3.26	3.04	3.05
Total body water (%)	M	54.43	54.87 ^bh^	54.37 ^b^	53.99 ^h^	54.43
	SD	4.35	3.84	3.73	3.79	3.77
Extracellular water (kg)	M	15.45 ^ef^	15.59 ^bh^	14.95 ^bej^	14.88 ^dhf^	15.39 ^dj^
	SD	1.74	1.83	1.85	1.78	1.85
Extracellular water (%)	M	45.27 ^ef^	45.29 ^bhi^	43.64 ^be^	43.34 ^dfh^	44.26 ^di^
	SD	2.59	3.10	3.77	3.34	3.48
Body cell mass (kg)	M	25.60 ^efg^	25.73 ^bhi^	26.80 ^be^	26.66 ^fh^	26.72 ^gi^
	SD	2.97	3.37	3.42	2.72	2.91
Body cell mass (%)	M	54.53 ^ef^	54.43 ^bhi^	56.66 ^be^	56.66 ^fh^	55.86 ^i^
	SD	3.21	4.19	3.84	3.29	3.58
Fat mass (kg)	M	16.49 ^af^	15.97 ^ahi^	16.33 ^c^	17.11 ^cdfh^	16.57 ^di^
	SD	6.12	5.37	5.31	5.64	5.43
Fat mass (%)	M	25.27 ^f^	24.71 ^h^	25.19 ^c^	26.06 ^cdfh^	25.17 ^d^
	SD	5.30	4.69	4.62	4.80	4.60
Intracellular water (%)	M	54.73 ^ef^	54.71 ^bhi^	56.36 ^be^	56.67 ^dfh^	55.74 ^di^
	SD	2.59	3.10	3.77	3.37	3.46
BMI [kg/m^2^]	M	21.47	21.38	21.29 ^j^	21.46	21.55 ^j^
	SD	2.90	2.72	2.64	2.72	2.68

M—mean; SD—standard deviation; statistically significant differences (*p* < 0.05) were marked: a—difference between 1 and 2; b—difference between 2 and 3; c—difference between 3 and 4; d—difference between 4 and 5; e—difference between 1 and 3; f—difference between 1 and 4; g—difference between 1 and 5; h—difference between 2 and 4; i—difference between 2 and 5; j—difference between 3 and 5. Numbers 1–5 mean successive periods of measurements.

**Table 2:** Statistical characteristics of the results of motor tests of female volleyball players in subsequent periods of the macrocycle.

Variables		Examinations				
		1	2	3	4	5
One-handed reach (cm)	M	225.74 ^efg^	226.22	226.36 ^e^	226.72 ^f^	226.57 ^g^
	SD	9.50	9.48	9.41	9.64	9.58
Two-handed reach (cm)	M	223.58 ^fg^	223.69 ^hi^	224.12	224.56 ^fh^	224.36 ^gi^
	SD	9.02	9.08	9.08	9.46	9.41
Standing vertical jump (cm)	M	262.52 ^aefg^	269.48 ^a^	268.30 ^ce^	269.99 ^cdf^	268.33 ^dg^
	SD	9.71	10.33	9.28	9.58	9.65
Running vertical jump (cm)	M	274.88 ^aefg^	279.95 ^ah^	280.41 ^ce^	283.64 ^cdfh^	280.79 ^dg^
	SD	10.19	10.04	9.42	10.47	9.73
Jumping without a run-up (cm)	M	38.94 ^aefg^	45.79 ^abi^	44.18 ^bcej^	45.43 ^cdf^	43.97 ^dgij^
	SD	5.23	7.80	6.32	6.29	6.45
Jumping with a run-up (cm)	M	49.15 ^aefg^	53.73 ^ah^	54.05 ^ce^	56.93 ^cdfh^	54.22 ^dg^
	SD	6.32	6.86	6.15	6.50	6.39
Medicine ball throw 2 kg (m)	M	8.15 ^f^	8.04 ^bhi^	8.32 ^b^	8.47 ^fh^	8.33 ^i^
	SD	1.10	1.27	1.16	1.03	1.01
Standing long jump (m)	M	1.87 ^aefg^	1.96 ^abhi^	2.03 ^be^	2.02 ^fh^	2.02 ^gi^
	SD	0.16	0.15	0.15	0.14	0.15

M—mean; SD—standard deviation; statistically significant differences (*p* < 0.05) were marked: a—difference between 1 and 2; b—difference between 2 and 3; c—difference between 3 and 4; d—difference between 4 and 5; e—difference between 1 and 3; f—difference between 1 and 4; g—difference between 1 and 5; h—difference between 2 and 4; i—difference between 2 and 5; j—difference between 3 and 5. Numbers 1–5 mean successive periods of measurements.

## Data Availability

The data presented in this study are available on request from the corresponding author.

## References

[1] Junior N.K.M. (2020). Specific periodization for the volleyball: The importance of the residual training effects. MOJ Sports Med..

[2] Albaladejo-Saura M., Vaquero-Cristóbal R., Marcos-Pardo P.J., Esparza-Ros F. (2021). Effect of an injury prevention program on the lower limb stability in young volleyball players. J. Sports Med. Phys. Fit..

[3] Bompa T.O., Haff G. (2009). Periodization: Theory and Methodology of Training.

[4] Pastuszak A., Buśko K., Kalka E. (2016). Somatotype and body composition of volleyball players and untrained female students—Reference group for comparison in sport. Anthropol. Rev..

[5] Wnorowski K. (2007). Relations between technical-tactical competence and speed-force skills in women volleyball players. Res. Yearbook.

[6] Pietraszewska J., Burdukiewicz A., Stachoń A., Andrzejewska J., Pietraszewski B. (2015). Anthropometric characteristic and lower limb power of professional female volleyball players. S. Afr. J. Res. Sport Phys. Educ. Recreat..

[7] Valleser C.W.M., Bersola K.A.R., Frances T.M., Papa E.L.V., Diaz F.C.B., Maghanoy M.L.A., Lariosa C.J.D. (2018). Anthropometric profile of elite women’s volleyball players in the Philippines. Turk. J. Kinesiol..

[8] Miftari F., Selimi M., Salihu H. (2018). Differences in some anthropometric parameters between basketball, handball and volleyball elite athletes in Kosovo. Sci. Hum. Kinet..

[9] Mielgo-Ayuso J., Calleja-González J., Clemente-Suárez V.J., Zourdos M.C. (2015). Influence of anthropometric profile on physical performance in elite female volleyballers in relation to playing position. Nutr. Hosp..

[10] Prokopec M., Padevětová M., Řemenář M., Železný J. (2003). Morpho-physiological characteristics of young female volleyball players. Pap. Anthropol..

[11] Duncan M.J., Woodfield L., al-Nakeeb Y. (2006). Anthropometric and physiological characteristics of junior elite volleyball players. Br. J. Sports Med..

[12] Teixeira D.M., del Fraro J., Soares F., Stanganelli L.C.R., Pires-Neto C.S., Petroski E.L. (2016). Anthropometric characteristic in elite athletes of the Brazilian team Juvenile and adult volleyball. Rev. Andal. Med. Deporte.

[13] Vujmilović A., Karalić T. (2014). Differences of body dimensions in female volleyball players (cadets) in relations to volleyball playing position. Sport J..

[14] Ostojic S.M. (2003). Seasonal alternations in body composition and sprint performance of elite soccer players. J. Exerc. Physiol. Online.

[15] Argus C.K., Gill N., Keogh J., Hopkins W.G., Beaven C.M. (2010). Effects of a short term pre-seson training programme on the body composition and anaerobic performance of professional rugby union players. J. Sports Sci..

[16] Bilsborough J.C., Greenway K., Livingston S., Cordy J., Coutts A.J. (2016). Changes in anthropometry upper body strength and nutrient intake in professional Australian Football Players During a season. Int. J. Sports Physiol. Perform..

[17] Requena B., García I., Suárez-Arrones L., Sáez de Villarreal E., Naranjo Orellana J., Santalla A. (2017). Off season effects on functional performance body composition and blood parameters in top level professional soccer players. J. Strength Cond. Res..

[18] Vuleta D., Jerak T., Sporiš G. (2016). Difference in jumping ability and body composition in competitive volleyball setters. Acta Kinesiol..

[19] Roelofs E.J., Smith-Ryan A.E., Trexler E.T., Hirsch K.R. (2017). Seasonal effects on body composition muscle characteristics and performance of collegiate swimmers and divers. J. Athl. Train..

[20] Barnes J.L., Schilling B.K., Falvo M.J., Weiss L.W., Creasy A.K., Fry A.C. (2007). Relationship of jumping and agility performance in female volleyball athletes. J. Strength Cond. Res..

[21] Perez-Gomez J., Rodriguez G.V., Ara I., Olmedillas H., Chavarren J., González-Henriquez J.J., Dorado C., Calbet J.A.L. (2008). Role of Muscle Mass on Sprint Performance: Gender Differences?. Eur. J. Appl. Physiol..

[22] Boldt M., Gregory D., Jaffe D., Dodge T.M., Jones M.T. (2011). Relationship between body composition and performance measures in NCAA Division III Women’s volleyball players. J. Strength Cond. Res..

[23] Nikolaidis P.T. (2013). Body mass index and body fat percentage are associated with decreased physical fitness in adolescent and adult female volleyball players. J. Res. Med. Sci..

[24] Ćopić N., Dopsaj M., Ivanović J., Nešić G., Jarić S. (2014). Body composition and muscle strength predictors of jumping performance: Differences between elite female volleyball competitors and nontrained individuals. J. Strength Cond. Res..

[25] Stamm R., Stamm M., Koskel S. (2002). Age, body build, physical ability, volleyball technical and psychophysiological tests and proficiency at competitions in young female volleyballers (aged 13–16 years). Pap. Anthropol..

[26] Nikolaidis P.T., Afonso J., Buśko K. (2014). Differences in athropometry, somatotype, body composition and physiological characteristics of female volleyball players by competition level. Sport Sci. Health.

[27] Malousaris G.G., Bergeles N.K., Barzouka K.G., Bayios I.A., Nassis G.P., Koskolou M.D. (2008). Somatotype, size and body composition of competitive female volleyball players. J. Sci. Med. Sport.

[28] Martín-Matillas M., Valdés D., Hernández-Hernández E., Olea-Serrano F., Sjöström M., Delgado-Fernández M., Ortega F.B. (2014). Anthropometric, body composition and somatotype characteristic of elite female volleyball players from the highest Spanish league. J. Sports Sci..

[29] Young K.C., Kendall K.L., Patterson K.M., Pandya P.D., Fairman C.M., Smith S.W. (2014). Rowing performance body composition and bone mineral density outcomes in college level rowers after season of concurrent training. Int. J. Sports Physiol. Perform..

[30] Mills C., Croix M.D.S., Cooper S.M. (2017). The Importance of Measuring Body Composition in Professional Football Players: A Commentary. Sports Exerc. Med..

[31] Stellingwerff T. (2018). Body composition periodization in an Olympic Level female middle distance runner over a 9 year career. Int. J. Sport Nutr. Exerc. Metab..

[32] Gonzalez A.M., Hoffman J.R., Rogowski J.P., Burgos W., Manalo E., Weise K., Fragala M.S., Stout J.R. (2013). Performance changes in NBA basketball players vary in starters vs. nonstarters over a competitive season. J. Strength Cond. Res..

[33] Milanese C., Piscitelli F., Lampis C., Zancanaro C. (2012). Effect of a competitive season on anthropometry and three compartment body composition in female handball players. Biol. Sport.

[34] Malý T., Malá L., Záhalka F., Baláš J., Čada M. (2011). Comparison of body composition between two elite women’s volleyball teams. Acta Gymnica.

[35] Hadzic R., Belica D., Popovic S. (2012). Comparative study of anthropometric measurement and body composition between elite basketball and volleyball players. Phys. Educ. Sport Health.

[36] Kutáč P., Sigmund M. (2017). Assessment of body composition of female volleyball players of various performance levels. J. Phys. Educ. Sport.

[37] Buśko K., Lipińska M. (2012). A comparative analysis of the anthropometric method and bioelectrical impedance analysis on changes in body composition of female volleyball players during the 2010/2011 season. Hum. Mov..

[38] Kavazis A.N., Wadsworth D.D. (2014). Changes in body composition and perceived stress scale-10 in National Collegiate Athletic Association Division I female volleyball players. Arch. Exerc. Health Dis..

[39] Stanforth P.R., Crim B.N., Stanforth D., Stults-Kolehmainen M.A. (2014). Body composition changes among female NCAA Division 1 atheletes across the competitive season and over a multiyear time frame. J. Strength Cond. Res..

[40] Pavlík J., Vespalec T., Zeman T. (2016). Change in body composition of female junior volleyball players. J. Hum. Sport Exerc..

[41] Stojanović T., Bešić D., Stojanović D., Lilić L., Zadražnik M. (2018). The effects of short term preseason combined training on body composition in elite female volleyball players. Anthropol. Noteb..

[42] Häkkinen K. (1993). Changes in physical fitness profile in female volleyball players during the competitive season. J. Sports Med. Phys. Fit..

[43] Manna I., Khanna G.L., Dhara P.C. (2011). Effect of Training on Anthropometric, Physiological and Health-Related Variables of Indian Senior Elite Volleyball Players. Asian J. Exerc. Sports Sci..

[44] Rousanoglou E.N., Barzouka K.G., Boudolos K.D. (2013). Seasonal changes of jumping performance and knee muscle strength in under-19 women volleyball players. J. Strength Cond. Res..

[45] Sheppard J.M., Newton R.U. (2012). Long-term training adaptations in elite male volleyball players. J. Strength Cond. Res..

[46] Sheppard J.M., Chapman D.W., Gough C., McGuigan M.R., Newton R.U. (2009). Twelve-month training-induced changes in elite international volleyball players. J. Strength Cond. Res..

[47] Sheppard J.M., Nolan E., Newton R.U. (2012). Changes in strength and power qualities over two years in volleyball players transitioning from junior to senior national team. J. Strength Cond. Res..

[48] Cosmin S.C., Mihaela R.A., Claudiu A. (2016). Anthropometric characteristics, body composition and physical performance of female cadet volleyball players. J. Phys. Educ. Sport.

[49] Konstantinos N.S., Panagiotis M.G., Ioannis B.A. (2019). Morphological characteristics of adolescent elite female handball and volleyball players. J. Phys. Educ. Sport.

[50] Newton R.U., Rogers R.A., Volek J.S., Häkkinen K., Kraemer W.J. (2006). Four weeks of optimal load ballistic resistance training at the end of season attenuates declining jump performance of women volleyball players. J. Strength Cond. Res..

[51] Nesser T.W., Demchak T.J. (2007). Variations of preseason conditioning on volleyball performance. J. Exerc. Physiol. Online.

[52] Stech M., Smulsky V. (2007). The estimation criteria of jump actions of high performance female volleyball players. Res. Yearbook.

[53] Mielgo-Ayuso J., Zourdos M.C., Calleja-González J., Urdampilleta A., Ostojic S.M. (2015). Dietary intake habits and controlled training on body composition and strength in elite female volleyball players during the season. Appl. Physiol. Nutr. Metab..

[54] Tsunawake N., Tahara Y., Moji K., Muraki S., Minowa K., Yukawa K. (2003). Body Composition and Physical Fitness of Female Volleyball and Basketball Players of the Japan Inter-high School Championship Teams. J. Physiol. Anthropol. Appl. Hum. Sci..

[55] Bozo D., Lleshi E. (2012). Comparison of Albanian female volleyball players with anthropometric, performance and haematological parameters. J. Hum. Sport Exerc..

[56] Polakovičová M., Vavák M., Ollé R., Lehnert M., Sigmund M. (2018). Vertical jump development in elite adolescent volleyball players: Effects of sex and age. Acta Gymnica.

[57] Kułaga Z., Litwin M., Tkaczyk M., Palczewska I., Zajączkowska M., Zwolińska D., Krynicki T., Wasilewska A., Moczulska A., Morawiec-Knysak A. (2011). Polish 2010 growth references for school-aged children and adolescents. Eur. J. Pediatr..

[58] Nikolaidis P.T., Ziv G., Arnon M., Lidor R. (2012). Physical Characteristics and Physiological Attributes of Female Volleyball Players-The Need for Individual Data. J. Strength Cond. Res..

[59] Lehnert M., Sigmund M., Lipińska P., Vařeková R., Hroch M., Xaverová Z., Stastny P., Háp P., Żmijewski P. (2017). Training-induced changes in physical performance can be achieved without body mass reduction after eight week of strength and injury prevention oriented programme in volleyball female players. Biol. Sport.

[60] Stamm R., Stamm M., Sorgina N., Koskel S. (2011). Training programme to develop young volleyballers’ jumping ability. Pap. Anthropol..

[61] Hyatt H.W., Kavazis A.N. (2019). Body Composition and Perceived Stress through a Calendar Year in NCAA I Female Volleyball Players. Int. J. Exerc. Sci..

[62] Ode J.J., Pivarnik J.M., Reeves M.J., Jeremy L.K. (2007). Body mass index as a predictor of percent fat in college athletes and nonathletes. Med. Sci. Sports Exerc..

[63] Klugland Torstveit M., Sundgot-Borgen J. (2012). Are under- and overweight female elite athletes thin and fat? A controlled study. Med. Sci. Sports Exerc..

[64] Talluri R., Liedtke E.I., Mohamed C., Maiolo R., Martinoli R., De Lorenzo A. (2003). The application of body cell mass index for studying muscle mass changes in health and disease conditions. Acta Diabetol..

[65] Heymsfield S.B., Wang Z.M., Baumgartner R.N., Ross R. (1997). Human body composition: Advances in models and methods. Annu. Rev. Nutr..

[66] Ronti T., Lupatelli G. (2006). The endocrine function of adipose tissue: An update. Clin. Endocrinol..

[67] Tomazo-Ravnik T., Jezernik D. (2008). Assessment of body composition of young female adults using anthropometry and bioelectric impedance analysis. Acta Med. Litu..

[68] Zaccagni L., Rinaldo N., Bramanti B., Mongillo J., Gualdi-Russo E. (2020). Body image perception and body composition: Assessment of perception inconsistency by a new index. J. Transl. Med..

[69] Tessutti L.S., Aguiar S.S., Costa G.D.C., Clemente F.M., Lima R.F., Neves R.V.P., Praça G.M., Castro H.O. (2019). Body composition and performance variables differences in female volleyball players by age group and playing position. Rev. Bras. Cineantropometria Desempenho Hum..

[70] González-Ravé J.M., Arija A., Clemente-Suarez V. (2011). Seasonal changes in jump performance and body composition in women volleyball players. J. Strength Cond. Res..

[71] Mielgo-Ayuso J., Collado P.S., Urdampilleta A., Martínez-Sanz J.M., Seco J. (2013). Changes induced by diet and nutritional intake in the lipid profile of female professional volleyball players after 11 weeks of training. J. Int. Soc. Sports Nutr..

[72] Pałka A. (2016). Changes in body mass composition of 14 year old female volleyball players of various rates of maturation. Sci. Rev. Phys. Cult..

[73] Roche A.F., Heymsfield S.B., Lohman T.G. (1996). Human Body Composition.

[74] Koley S., Singh J., Sandhu J.S. (2010). Anthropometric and physiological characteristics on Indian inter-university volleyball players. J. Hum. Sport Exerc..

[75] Bankovic V., Dopsaj M., Terzic Z., Nesic G. (2018). Descriptive Body Composition Profile in Female Olympic Volleyball Medalists Defined Using Multichannel Bioimpedance Measurement: Rio 2016 Team Case Study. Int. J. Morphol..

[76] Makiel K., Suder A., Kasza S., Kubasiak K. (2020). Body composition and dietary patterns in professional and amateur bodybuilders. Anthropol. Rev..

[77] Nadel E.R. (1979). Control of sweating rate while exercising in the heat. Med. Sci. Sports Exerc..

[78] Cheuvront S.N., Kenefick R.W. (2017). Improving the status quo for measuring whole body sweat losses. J. Appl. Physiol..

[79] Silva A.M., Matias C.N., Santos D.A., Rocha P.M., Minderico C.S., Sardinha L.B. (2014). Increases in Intracellular Water Explain Strength and Power Improvements Over a Season. Int. J. Sports Med..

[80] Katic R., Grgantov Z., Jurko D. (2006). Motor structures in female volleyball players aged 14–17 according to technique quality and performance. Coll. Antropol..

[81] Tsoukos A., Drikos S., Brown L.E., Sotiropoulos K., Veligekas P., Bogdanis G.C. (2019). Anthropometric and Motor Performance Variables are Decisive Factors for the Selection of Junior National Female Volleyball Players. J. Hum. Kinet..

[82] Bilici Ö.F., Selçuk M. (2018). Evaluation of the Effect of Core Training on the Leap Power and Motor Characteristics of the 14–16 Years Old Female Volleyball Players. J. Educ. Train. Stud..

[83] Collins S.M., Silberlicht M., Perzinski C., Smith S., Davidson P. (2014). The relationship between body composition and preseason performance tests of collegiate male lacrosse players. J. Strength Cond. Res..

[84] Carter J.G., Potter A.W., Brooks K.A. (2014). Overtraining syndrome: Causes, consequences, and methods for prevention. J. Sport Hum. Perf..

[85] Gamble P. (2006). Periodization of training for team sports athletes. Strength Cond. J..

[86] Sale D.G. (1988). Neural adaptation to resistance training. Med. Sci. Sports Exerc..

[87] Gabbett T., Georgieff B., Anderson S., Cotton B., Savovic D., Nicholson L. (2006). Changes in skill and physical fitness following training in talent-identified volleyball players. J. Strength Cond. Res..

[88] Acar H., Eler N. (2019). The Relationship between Body Composition and Jumping performance of Volleyball Players. J. Educ. Train. Stud..

[89] Aslan C.S., Büyükdere C., Köklü Y., Özkan A., Özdemir N.Ş. (2011). The relationships among body composition, anaerobic performance and back strength characteristics of sub-elite athletes. J. Hum. Sci..

[90] Aouichaoui C., Trabelsi Y., Tabka Z., Dogui M., Richalet J., Bouhlel E. (2014). Effect of anthropometric characteristics and socio-economic status on vertical jumping performances in Tunisian athletic children. Am. J. Sports Med..

[91] Morrow J.R., Jackson A.S., Hosler W.W., Kachurik J.K. (1979). The importance of strength, speed, and body size for team success in women’s intercollegiate volleyball. Res. Q. Am. Assoc. Health Phys. Educ..

